# Leaching of Columbite Using an Organic Acid to Obtain Nb and Ta: Effects of Sulfate Thermal Treatment on Hydrometallurgical Processing

**DOI:** 10.1021/acsomega.5c11374

**Published:** 2026-03-17

**Authors:** Danielly Cristina da Silva Lucio, Thamyres Cardoso de Carvalho, Bárbara da Rocha Pereira, Denise Crocce Romano Espinosa, Jorge Alberto Soares Tenório, Amilton Barbosa Botelho Junior

**Affiliations:** † Department of Chemical Engineering, Polytechnical School, 28133 University of São Paulo, São Paulo, SP 05508-080, Brazil; ‡ Department of Chemical Engineering, 42511 FEI University, São Bernardo do Campo, SP 09850-901, Brazil; § Department of Chemical Engineering, 8018 Norwegian University of Science and Technology, Trondheim 7491, Norway

## Abstract

Global energy transition is driving the search for alternative, sustainable methods for producing critical minerals. This study evaluates thermal treatments of columbite, followed by organic acid leaching to recover Nb and Ta. Hydrometallurgical processes currently use HF combined with other inorganic acids for columbite leaching, and here, the use of oxalic acid after sulfation. Our analysis considers KHSO_4_ a better technical alternative than H_2_SO_4_, achieving 90% leaching efficiency. Leaching of Nb and Ta by oxalic acid (H_2_C_2_O_4_) after thermal treatment is possible due to the formation of stable metal-oxalate complexes, avoiding risks (operational and occupational health) of handling HF leaching. Leaching efficiencies reached 92.8% for Nb (3.48g/L) and 88.4% for Ta (0.45g/L) at a L/S ratio of 3 (columbite/KHSO_4_) at 650 °C for 3 h, followed by leaching with 1.0 mol/L H_2_C_2_O_4_ at a L/S ratio of 10 at 90 °C for 8 h. However, a preliminary economic analysis indicated that the proposed process is currently not economically feasible, mostly due to the cost of organic acid.

## Graphical abstract

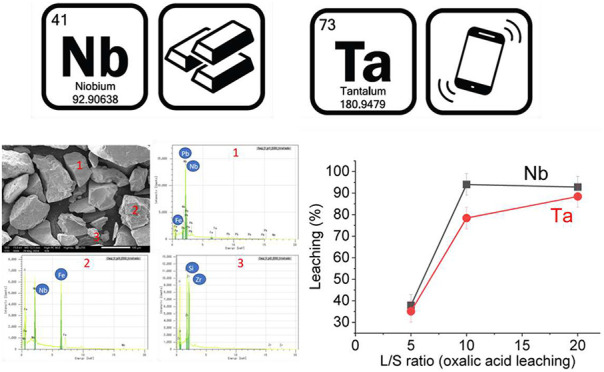

## 1 Introduction

Nb and Ta are critical raw materials for energy transition for the production of iron and ferroalloy metals, 3D printing, electronic capacitors, and digital technologies. About 92% of Nb supply to the European Union is from Brazil, while 35% of the Ta supply is from the Democratic Republic of Congo.^1–3^ Pyrochlore, (Na,Ca)_2_Nb_2_O_6_(OH,F), is the main Nb-bearing mineral that is a commercial primary source, while columbite, (Fe,Mn)­Nb_2_O_6_, is the second most common Nb-bearing mineral. Other secondary sources (e.g., Sn slag) are being explored.^4,5^ However, interest in columbite mineral has increased due to the high Nb content (30–75% Nb_2_O_5_ and 1–40% Ta_2_O_5_), along with Fe (0.1%–18%) and Mn as ferrocolumbite, manganocolumbite, magnesiumcolumbite, ferrotantalite, and coexisting elements such as MnO, Mg, U, and Th.^4,6,7^

The process of extraction of Nb and Ta from these minerals has been described in the literature using inorganic acids such as HF or H_2_SO_4_. The difficulties in extracting Nb and Ta are also related to the amount of impurities present in the mineral and the generation of toxic effluents, but mostly to the complexity of bearing minerals. There are three routes used to extract Nb and Ta from columbite: pyrometallurgical, hydrometallurgical, or the hybrid (pyro + hydro) route.^6,8,9^

Pyrometallurgical processing techniques include carbothermic reduction (reduction of metal oxides using C as a reducing agent) and aluminothermic reduction (a redox reaction that uses Al at temperatures above 1,000 °C and reduces metal oxides).^6^ In direct leaching, HF is mostly used due to its corrosion capability; then, fractional crystallization occurs for Nb/Ta separation. HCl or H_2_SO_4_ is added to reduce the use of HF, followed by solvent extraction (MIBK or TBP).^10–13^ Alternatives ((pyro+hydro) route) are being investigated to reduce carbon footprint and environmental impacts, and hydrometallurgical processing plays a pivotal role in meeting the *sustainable mining goals*.^14,15^

Pretreatment with acids or alkalis (e.g., NaOH, KOH, KHSO_4_, or H_2_SO_4_) prior to water/acid leaching has been used to convert columbite into soluble compounds. The use of KHSO_4_ (sulfation) for columbite pretreatment was proposed to form soluble sulfates as an alternative to H_2_SO_4_, which is used to remove impurities such as Fe and Mn present in the mineral, in addition to forming soluble Nb and Ta compounds.^6,16,17^ Previous studies from our group reported low leaching efficiency on sulfation (H_2_SO_4_ and KHSO_4_), followed by water leaching (<40%), but higher efficiencies were observed for oxalic acid (H_2_C_2_O_4_) leaching in our group (>90%).^5,18–20^

Sulfation of columbite by KHSO_4_, followed by water leaching, allows partially removal of impurities, while Nb/Ta remains in the solid phase^21^; and then, leaching of Nb (94%) and Ta (75%) of Sn slag by H_2_C_2_O_4_ was reported. No literature was found reporting the H_2_C_2_O_4_ leaching of columbite after sulfation, only Sn slag from our group^18^ and a combination of H_2_C_2_O_4_–HNO_3_.^22^ As industries use H_2_SO_4_ for sulfation, a comparison is necessary, including technical and economic feasibilities.

In this study, columbite from a Brazilian site was studied to evaluate the impact of sulfation by KHSO_4_ and compared to that by H_2_SO_4_ as a pretreatment. Acid leaching experiments after thermal treatment were performed with H_2_C_2_O_4_ and H_2_SO_4_. Preliminary economic analysis was considered for decision-making regardless of technical feasibility. This work represents advances in the search for hydrometallurgical alternatives for the extraction of critical metals.

## 2 Materials and Methods

### 2.1 Materials

The columbite sample used was provided by a Brazilian mining company, whose primary production line is metallic Sn. However, there is also secondary production of the so-called columbite, which constitutes the basis of our study. Initially, the ore is extracted at the mine, followed by the stages of crushing and recrushing; subsequently, milling is carried out, which increases the material’s surface area and prepares it for the concentration stage, during which a preconcentrated material is obtained. This preconcentrate is then sent to the stages of tin flotation, mixed flotation, and niobate flotation, the latter being the stage that concentrates the minerals bearing Nb and Ta, from which the sample is derived. The chemical characterization and the thermal treatment/leaching experiments were conducted using analytical-grade (P.A.) reagents, including ultrapure water.

### 2.2 Methods

#### 2.2.1 Sample Characterization

The sample homogenization was performed by a conical pile technique and later using a Jones type in portions of 250 g. Laser granulometric analysis was conducted to determine the particle-size distribution using ultrapure water (Mastersizer 2000, Malvern Instruments). To determine the morphology and elemental chemical composition of the sample, the sample was dried in a drying oven for 24 h at 60 °C and coated with Au, and the analysis was performed using scanning electron microscopy coupled with energy-dispersive X-ray spectroscopy (SEM-EDS, JCM 7000, JEOL). For mineralogical analysis, the sample was ground until a talc-like texture was achieved, and X-ray diffraction (XRD) analysis was performed from 20 to 80 °C in 0.02° steps and a scanning speed of 6.5°/min (MiniFlex 300, Rigaku).

For characterization via fusion (in triplicate), a mixture of lithium tetraborate (Li_2_B_4_O_7_) and lithium metaborate (LiBO_2_) in a 66.67%/33.33% ratio, along with 0.5% lithium bromide (LiBr), was used. The material was mixed in a platinum crucible with 0.7 g of the sample and 7 g of flux for fusion (Li_2_B_4_O_7_ + LiBO_2_ + LiBr) and heated at 1,200 °C for 40 min under constant agitation. After cooling (room temperature), the resulting material was ground and 0.2 g was added to a beaker with 20 mL of HCl P.A. and stirred for 10 min at 100 °C. Dilutions were prepared in 3% HNO_3_ and analyzed by inductively coupled plasma mass spectrometry (Shimadzu 2030 (ICP-MS)) for the determination of rare earth elements and inductively coupled plasma optical emission spectrometry (ICP-OES) on the Agilent 700 series and EDX Shimadzu 7000 equipment for the determination of other elements.

#### 2.2.2 Leaching Experiments

The columbite pretreatment was performed with potassium bisulfate (KHSO_4_) and sulfuric acid (H_2_SO_4_). Leaching experiments were performed with oxalic acid (H_2_C_2_O_4_).

To optimize the recovery of and from columbite, two approaches were compared: direct leaching with H_2_C_2_O_4_ and leaching following thermal pretreatment using KHSO_4_ in a muffle furnace^23^ or H_2_SO_4_.^24^ H_2_C_2_O_4_ was chosen due to its ability to form soluble complexes with these metals. The thermal pretreatment was employed to modify the mineral structure of the columbite, facilitating the formation of soluble compounds for subsequent leaching.^5,18,19,24^

Leaching experiments were performed using a three-neck reactor placed on a heating plate, coupled with a condenser connected to a thermostatic bath set at 0 °C. For experiments without pretreatment, the solid-to-liquid ratio (S/L), acid concentration, temperature, and time (Table S1) were evaluated.

For the pretreatment tests using KHSO_4_, the sample and KHSO_4_ were homogenized, placed in a ceramic crucible, and heated in a muffle furnace at 650 °C for 3 h.^24^ The solid-to-solid (S/S) ratios evaluated were 1/3, 1/5, 1/7, and 1/10. Then, the crucibles were removed from the furnace, and the treated mixtures were ground and weighed for the leaching stage using H_2_C_2_O_4_. During leaching, the acid concentration, temperature, and time were fixed at 1 mol/L, 90 °C, and 8 h, respectively, while the S/L ratios were evaluated at 1/5, 1/10, and 1/20.

For the pretreatment experiments using H_2_SO_4_, the sample and H_2_SO_4_ were placed in a ceramic crucible and heated in a muffle furnace for 2 h.^18,19^ The S/L ratios evaluated were 1/3, 1/5, 1/7, and 1/10, while the temperature was varied at 250 °C, 350 °C, and 450 °C. Then, the crucibles were removed, and the treated mixtures were ground and weighed for the leaching stage using H_2_C_2_O_4_. During this stage, the S/L ratio, temperature, and time were fixed at 1/5, 90 °C, and 8 h, respectively, while acid concentration (0.1, 0.5, and 1.0 mol/L) was evaluated. The leaching solutions were vacuum-filtered, diluted with HNO_3_ 3%, and analyzed by ICP-OES for mass balance.

## 3 Results and Discussion

### 3.1 Sample Characterization

The particle-size distribution analysis of the columbite sample revealed that 90% of the particles (D90) have diameters equal to or smaller than 154.8 μm, 50% (D50) are below 79.7 μm, and 10% (D10) do not exceed 37.3 μm (Figure 1). This homogeneous distribution is predominantly composed of particles under 150 μm and is associated with a dark coloration.

**Figure 1:**
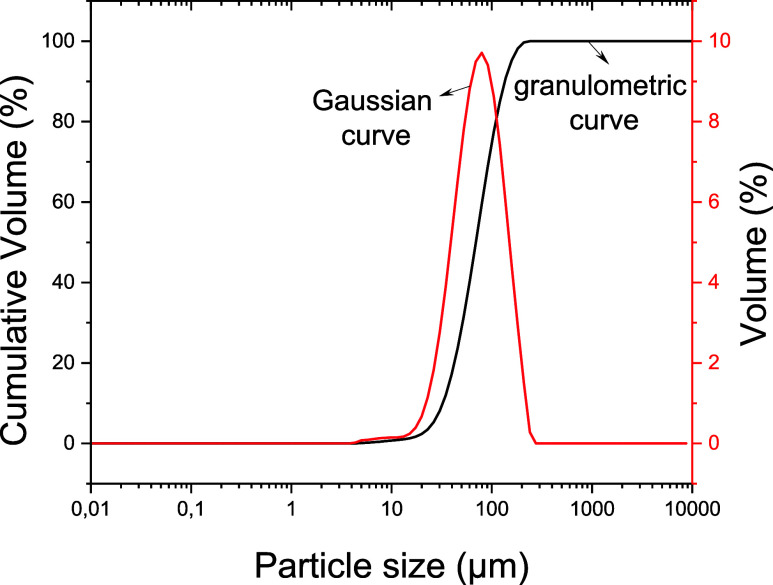
Particle-size distribution of the columbite sample used in this study.

The Gaussian curve exhibits a unimodal distribution characterized by a single peak, indicating uniformity in particle size as a result of the applied processing. Additionally, the narrow base of the curve suggests low particle-size dispersion, as evidenced by the difference between D90 and D10, indicating that most of the particles have similar sizes (Rajak et al.^22^).

Allou (2019) observed a heterogeneous and bimodal profile of the Ivory Coast’s columbite–tantalite and a diameter of 7.6 mm. In contrast, the sample analyzed in our study exhibits smaller and more uniform particles, attributed to mineral beneficiation applied prior to characterization. Nzeh et al.^8^ observed an average particle size ranging from 250 to 355 μm for columbite samples from Nigeria.

The diffractogram of the analyzed columbite sample (Figure 2) reveals the presence of FeNb_2_O_6_ (ferrocolumbite), SiO_2_ (quartz), UO_2_ (uranium oxide), and Pb_1.5_Nb_2_O_6.5_ (pyrochlore), as similarly reported (Brune et al., 2008; Dang et al., 2022) for columbites characteristic of the Amazon region of Brazil associated with albite and pegmatites (Hadlich et al., 2019). Lv and Zihu (2022) exhibited a distinct mineralogical composition of columbite, predominantly consisting of Quartz (SiO_2_), Topaz (Al_2_SiO_4_(F,OH)_2_), Sphalerite (ZnS), Galena (PbS), Albite (NaAlSi_3_O_8_), and Orthoclase (KAlSi_3_O_8_), as also found in minerals typically found in China.

**Figure 2:**
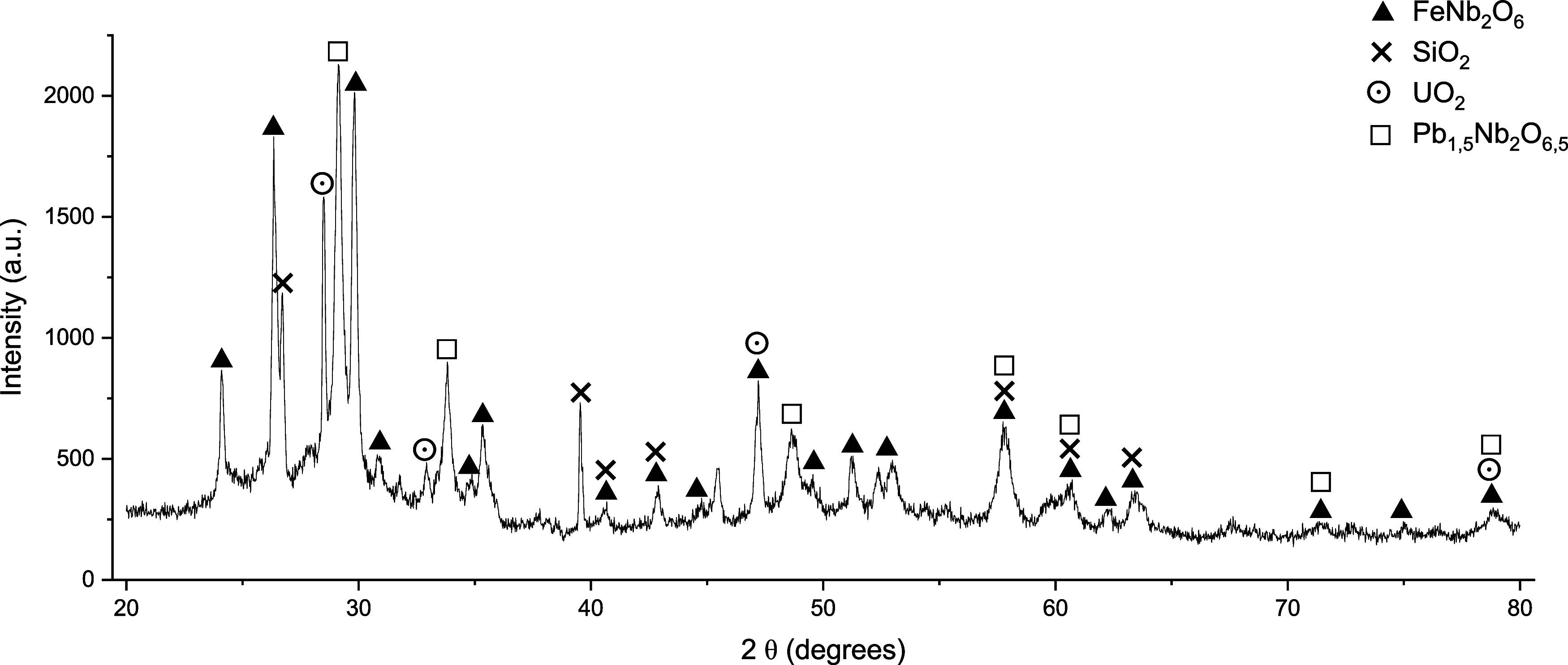
X-ray diffractometry of columbite sample showing the main phases (ICSD: FeNb_2_O_6_, 201962; SiO_2_, 00–078–1253; UO_2_, 246851; Pb_1.5_Nb_2_O_6.5_, 031318).

The SEM micrographs (Figure 3) showed the morphology of the columbite sample particles. The analyses were conducted using secondary electrons, highlighting particles with different sizes and degrees of surface roughness. The highlighted regions were analyzed by EDS for elemental identification, and the corresponding spectra indicate the presence of Nb, Pb, Fe, Si, Zr, F, Al, and Na. The elements C and Au were excluded from the interpretation as they originate from the sample holder and the metallic coating, respectively.

**Figure 3:**
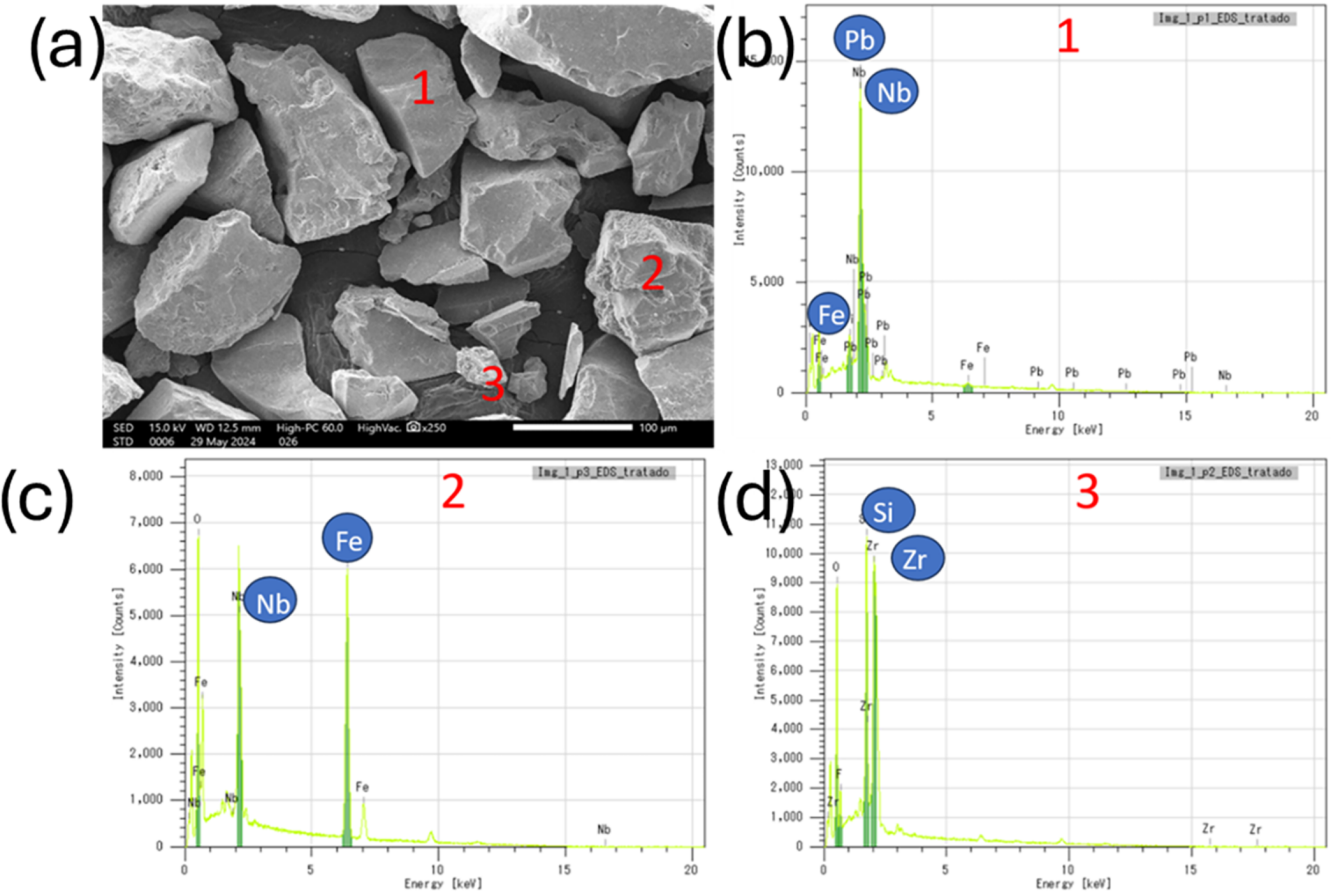
(a) SEM images of columbite (Region 1) and (b–d) the corresponding EDS spectra of the particles indicated by the numbers.

Small, rough particles (e.g., Figure 3, Particle 3) predominantly contain Si and Zr. In contrast, large, rough particles (e.g., Figure 3, Particle 2) exhibit high concentrations of Nb, Fe, and Pb. Conversely, large, smooth particles (e.g., Figure 3, Particle 1) are composed of Nb, Fe, and Si. Collectively, these results indicate the mineralogical heterogeneity of the sample, confirming the presence of ferrocolumbite, Pb phases associated with pyrochlore, and other minerals containing Zr and Si.

The identified elemental composition (Table 1) is consistent with previous studies that reported the occurrence of Th, Zr, Pb, Fe, Hf, Y, U, Nb, and Ta in columbite samples from the Amazon region.^25^ However, the material analyzed underwent a flotation process, which may have reduced the concentrations of Th and Zr.^8^

**Table 1:** Columbite Composition by a Fusion Method Using a Mixture of Li_2_B_4_O_7_, LiBO_2_, and LiBr in HCl P.A., Followed by Analysis in ICP-OES and ICP-MS

element	concentration (%)	oxide	concentration (%)
Nb	22.65	Nb_2_O_5_	32.4
Ta	3.04	Ta_2_O_5_	3.71
Fe	8.62	Fe_2_O_3_	12.33
Pb	12.58	PbO	13.55
Si	3.59	SiO_2_	7.68
U	4.74	U_3_O_8_	5.59
Th	3.42	ThO_2_	3.9
Zr	2.21	ZrO_2_	2.99
Hf	0.28	HfO_2_	0.32
Mn	1.17	MnO	1.51
Sn	2.41	SnO_2_	3.05
Zn	0.39	ZnO	0.48
Al	0.58	Al_2_O_3_	1.09
Ti	0.3	TiO_2_	0.5
Na	1.72	Na_2_O	2.32
Ca	3.67	CaO	5.14
K	1.25	K_2_O	1.51
P	0.14	P_2_O_5_	0.63
Mg	2.2	MgO	3.29
Ce	1	CeO_2_	1.23
Y	0.58	Y_2_O_3_	0.74
Dy	0.35	Dy_2_O_3_	0.41
La	0.26	La_2_O_3_	0.31
Nd	0.34	Nd_2_O_3_	0.39
Pr	0.11	Pr_6_O_11_	0.13
Gd	0.12	Gd_2_O_3_	0.14
Er	0.17	Er_2_O_3_	0.2
Tb	5	Tb_4_O_7_	0.05
Yb	0.12	Yb_2_O_3_	0.14
Ho	0.05	Ho_2_O_3_	0.06
Eu	0.002	Eu_2_O_3_	0.002
Lu	0.01	Lu_2_O_3_	0.01
Sc	0.01	Sc_2_O_3_	0.02
Sm	0.17	Sm_2_O_3_	0.2
Tm	0.02	Tm_2_O_3_	0.02

Nb and Ta account for about 23% and 3% of the columbite sample, respectively. Nb and Ta concentrations were reported as 46% and 6%, respectively, containing zircon, hematite, cassiterite, columbite, and pyrochlore, without physical separation.^26^ Other samples were reported containing 10% Ta and 4% Nb.^27^ Nb and Ta concentrations (Table 1) slightly differ from those reported in the literature (as reported in ref 26 and in ref 27), which may be related to the geological location of the deposit and to the physical beneficiation processes applied before sample collection, as reported in the Introduction section.

### 3.2 Leaching Experiments

These experiments aim to evaluate the effect of thermal treatment with KHSO_4_ or H_2_SO_4_ on the subsequent H_2_C_2_O_4_ leaching of Nb and Ta from columbite. While direct leaching and thermodynamic simulations have been reported previously,^5,18^ we focus here on bridging the knowledge gap regarding how sulfate-based thermal pretreatment influences the efficiency of oxalic acid leaching. Equations 1–6 were developed based on our previous developments^5,18,19,24^ and conclusions of our experiments.

#### 3.2.1 Direct Leaching with H_2_C_2_O_4_

Low leaching rates of Nb and Ta (Figure 4) were observed in the direct leaching reaction due to the strong acid resistance of FeNb_2_O_6_ and Pb_1.5_Nb_2_O_6.5_ ^10,22,23,28–30^ ; however, our data reports better results using organic acid (H_2_C_2_O_4_) than inorganic acid (as H_2_SO_4_, as also shown in Table S2).^24^ Based on Nb/Ta–H_2_O Pourbaix Diagram,^5^ Nb oxide can be dissolved in solution with pH below 0.5, but a comparison with the literature has demonstrated that H_2_C_2_O_4_ might have higher kinetic leaching than inorganic acids, resulting in better leaching rates.^18,22,24^ Compared to H_2_SO_4_ (Table S2), leaching of Nb and Ta was lower than 1.0%; however, the leaching could be explored in future processes for impurities removal, such as Fe (49.4%), U (22.1%), and Th (70.5%), depending on economic feasibility (considering separation/purification steps). Therefore, thermal treatment (sulfation) transforms Nb and Ta compounds into better soluble compounds^21^ and avoids the use of fluoride compounds.^19^

**Figure 4:**
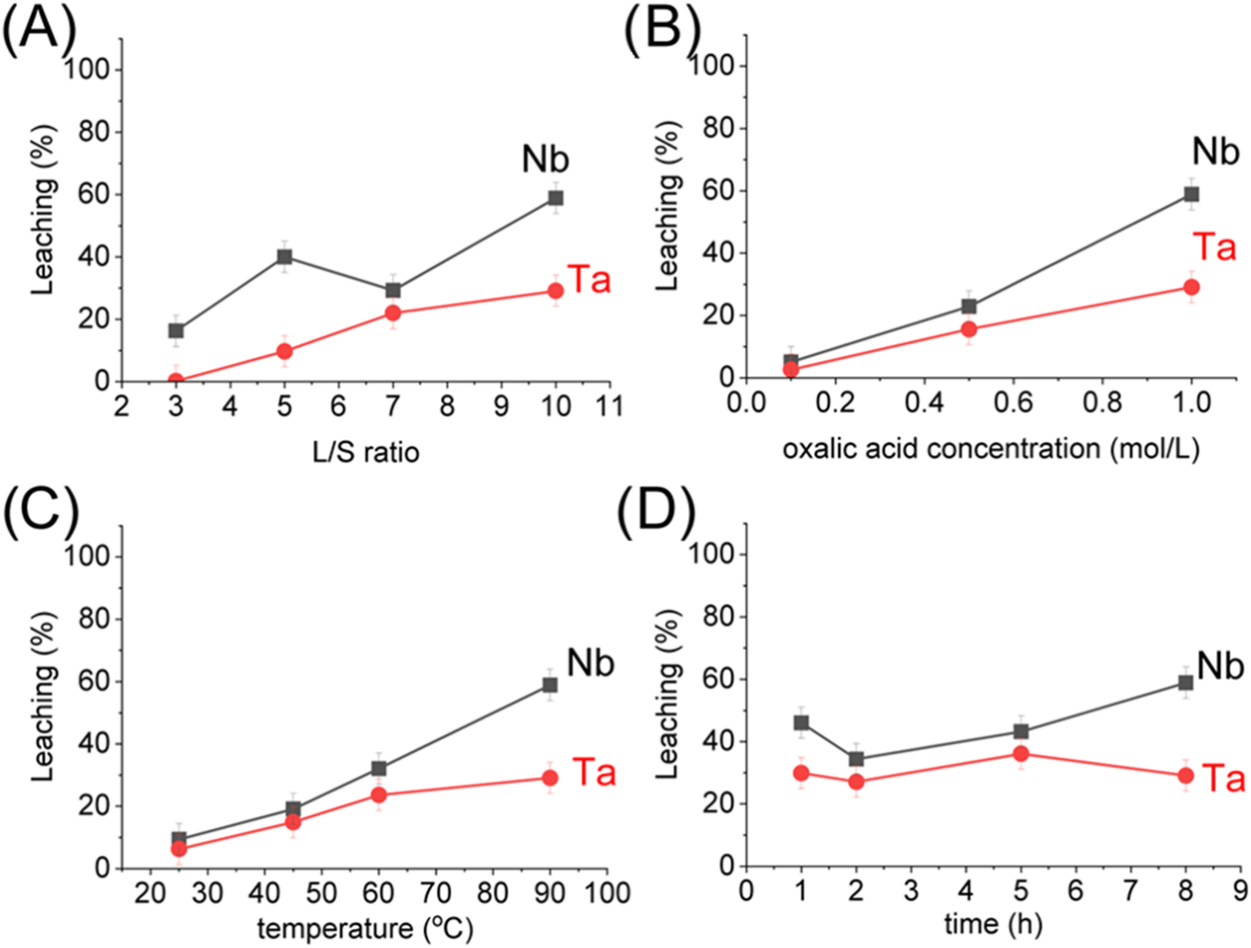
(a) Effect of the L/S ratio on the direct leaching of columbite with 1.0 mol/L H_2_C_2_O_4_ for 8 h at 90 °C. (b) Effect of H_2_C_2_O_4_ concentration on the direct leaching of columbite, at a L/S ratio of 10, for 8 h at 90 °C. (c) Effect of temperature on the direct leaching of columbite with 1.0 mol/L H_2_C_2_O_4_ at a L/S ratio of 10 for 8 h. (d) Effect of time on the direct leaching of columbite with 1.0 mol/L H_2_C_2_O_4_ at a L/S ratio of 10 at 90 °C for 8 h.

An increase in the leaching of Nb and Ta was observed as the L/S ratio decreased (a proportional increase in the amount of acid available for reaction; Figure 4a) owing to the greater availability of H_2_C_2_O_4_ to react with Nb and Ta. The higher acid concentration (relative to a solid) promotes the formation of soluble complexes (Table 2). Although low leaching efficiency may indicate low kinetic or thermodynamic driving force, our data demonstrate the opposite (Figure 4c,d), which might be related to compounds present (FeNb_2_O_6_ and Pb_1.5_Nb_2_O_6.5_) in columbite due to their refractory properties, resulting in low efficiency on direct leaching. Increasing the temperature enhances the reaction kinetics between H_2_C_2_O_4_ and the refractory metals Nb and Ta, thus promoting the formation and stability of the metal–oxalate complexes. An additional possibility that limits the leaching efficiencies, in addition to the refractory nature of the material, is the formation of a passivation layer,^18,22,24^ and clarification about the mechanism of its formation and composition can be explored in future studies. Maximum leaching of Nb and Ta reached 60% and 25%, respectively, at a L/S ratio of 10, acid concentration of 1.0 mol/L at 90 °C for 8 h. Leaching of other metals (Table S2) also increased with the increase of the L/S ratio, where Fe (96.3%), U (89.5%), and Hf (25.0%) are the most leached. It is expected that as the L/S ratio increases, the proportion of acid per solid material increases, resulting in an excess of acid available for leaching. Machaca et al. observed that an excess of over 50g/L of the S/L ratio results in precipitation of Nb and Ta.^18^ Therefore, we can assume that our experiments do not result in losses of Nb and Ta.

**Table 2:** Main Complexes Formed between Nb and Ta with H_2_C_2_O_4_

[NbO(C_2_O_4_)_3_]^3–^	^29^
[NbO(C_2_O_4_)_2_(H_2_O)_2_]^−^	^30^
[NbO(OH)(C_2_O_4_)_2_]_2_^–^	^29,30^
[TaO(C_2_O_4_)_3_]_3_^–^	^28^
[TaOH(C_2_O_4_)_3_]_2_^–^	^29^
[Ta_4_O_8_(C_2_O_4_)_3_]_2_^–^	^29,30^

#### 3.2.2 Leaching with H_2_C_2_O_4_ after Pretreatment with KHSO_4_

The thermal treatment used is aimed at converting refractory Nb and Ta minerals into more soluble compounds, thereby facilitating their subsequent leaching. During heating at temperatures above 400 °C, KHSO_4_ acts as an oxidizing flux, and it is transformed into potassium pyrosulfate (K_2_S_2_O_7_) (eq 1) and then decomposes, releasing sulfur trioxide (SO_3_) (eq 2). The generated SO_3_ reacts with Nb and Ta oxides present in ferrocolumbite, forming soluble acidic sulfates such as NbO­(SO_4_) and TaO­(SO_7_) (eqs 3 and 4).

1 $$2{\mathrm{KHSO}}_{4}\to 2K^{+}+H_{2}O+S_{2}O_{7}^{2-}\to H_{2}O↑+K_{2}S_{2}O_{7}$$

2 $$K_{2}S_{2}O_{7}\to K_{2}O+2SO_{3}↑$$

3 $$Nb_{2}O_{5}+5SO_{3}\to 2\mathrm{NbO}(SO_{4}{)}_{2}$$

4 $$Ta_{2}O_{5}+5SO_{3}\to 2\mathrm{TaO}(SO_{4}{)}_{2}$$

The leaching of Nb and Ta increases with the amount of KHSO_4_ added to columbite during the thermal treatment (Figure 5A). The columbite/KHSO_4_ ratio of 1:3 results in the highest leaching efficiency with H_2_C_2_O_4_, reaching 94% for Nb and 80% for Ta. This indicates that in this case, even lower proportions of KHSO_4_ are sufficient to promote the formation of soluble sulfates, which are subsequently complexed during leaching with H_2_C_2_O_4_.

**Figure 5:**
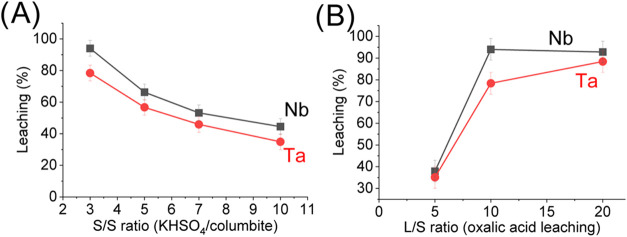
(A) Effect of the S/S ratio between columbite and KHSO_4_ during thermal treatment for 3 h at 650 °C on the leaching with 1.0 mol/L H_2_C_2_O_4_ at a L/S ratio 10 for 8 h at 90 °C. (B) Effect of the L/S ratio of the treated sample/acid on leaching with 1.0 mol/L H_2_C_2_O_4_ for 8 h at 90 °C, after pretreatment with KHSO_4_ at an S/S ratio of 3 for 3 h at 650 °C.

During the leaching step, soluble sulfates of Nb and Ta react with oxalate (C_2_O_4_^2–^), forming stable and soluble oxalate complexes such as [NbO­(C_2_O_4_)_3_]^3–^ and [TaO­(C_2_O_4_)_3_] ^3–^ (eqs 5 and 6).^18^

5 $$\mathrm{NbO}\left(SO_{4}\right)+3H_{2}C_{2}O_{4}\to H_{3}[\mathrm{NbO}\left(C_{2}O_{4}{)}_{3}\right]+H_{2}SO_{4}$$

6 $$\mathrm{TaO}\left(SO_{4}\right)+3H_{2}C_{2}O_{4}\to H_{3}[\mathrm{TaO}\left(C_{2}O_{4}{)}_{3}\right]+H_{2}SO_{4}$$

After the thermal treatment conditions with KHSO_4_ were established at an S/S ratio of 1:3 at 650 °C for 3 h, the influence of the S/L ratio during leaching with H_2_C_2_O_4_ was evaluated with the aim of assessing whether the leaching percentage of the metals varied with the amount of H_2_C_2_O_4_ added. The S/L ratios of 1/5, 1/10, and 1/20 were tested (Figure 3(b), and it was observed that the extraction of Nb and Ta increased with greater availability of H_2_C_2_O_4_, indicating enhanced dissolution of the metal sulfates formed during the thermal treatment. Compared to H_2_SO_4_ leaching (Figure S1), organic acid leaching (H_2_C_2_O_4_) showed the best results due to Nb- and Ta-based complex formation. Inorganic acid leaching (H_2_SO_4_) reached 17.2% for Nb and 2.3% for Ta. Excess sulfate ions are not beneficial for columbite leaching (a combination of sulfate roasting (KHSO_4_) and sulfate leaching (H_2_SO_4_)), decreasing the leaching of metals. It can be claimed that low leaching efficiencies were observed for Ta and Nb in both direct leaching and after thermal treatment with sulfate-based compounds, where Nb leaching was less than 20% and Ta leaching was less than 5% for direct leaching and less than 5% for Nb and close to zero for Ta after thermal treatment. However, Nb leaching could be reached using a strong acid solution as a combination of H_2_SO_4_ (10.8 mol/L) and HNO_3_ (5.3 mol/L) at 200 °C^31^; however, in the solution after leaching, it is reported that Nb precipitates even at pH 0.2,^31–33^ and 8–12 mol/L H_2_SO_4_ is reported for mineral decomposition.^5^ In our study, we were looking for mild conditions for the leaching of Nb and Ta from columbite.

#### 3.2.3 Leaching with H_2_C_2_O_4_ after Pretreatment with H_2_SO_4_

In the H_2_SO_4_ thermal treatment, columbite is converted from refractory oxides (or related compounds such as FeNb_2_O_6_ and Pb_1.5_Nb_2_O_6.5_, as shown in Figure 2) into Nb and Ta into soluble compounds. During heating, H_2_SO_4_ undergoes thermal decomposition, releasing SO_3_, which reacts with Nb- and Ta-bearing compounds and converts them into sulfates. However, in contrast to KHSO_4_ roasting (eqs 1–4), H_2_SO_4_ is aqueous and already interacts with columbite, reacting and rapidly increasing with the temperature. Although H_2_SO_4_ shows low acid consumption and faster kinetics, the concentrated acid raises safety concerns, and the analysis of H_2_SO_4_ on sulfation was motivated by the large literature and industrial knowledge, while solid salt as a sulfating agent allows greater physical contact with the ore particles and minimizes losses due to volatilization.^34–38^ In our experiments, H_2_C_2_O_4_ leaching was performed after H_2_SO_4_ sulfation. H_2_SO_4_ leaching experiments were performed, and the results are shown in Figure S2.

The increase in the amount of H_2_SO_4_ in sulfation (thermal treatment) has almost no effect in Nb and Ta leaching (Figure 6A), but reached higher leaching efficiencies than direct leaching by H_2_SO_4_ (Table S2) and lower than H_2_C_2_O_4_ direct leaching (Figure 4). Sulfate-based compounds formed in sulfation reacted with H_2_C_2_O_4_ and formed soluble oxalate-based compounds (eqs 5 and 6) and benefited the leaching of Nb and Ta. Temperature (Figure 6B) is an important parameter in sulfation due to the H_2_SO_4_ boiling point (337 °C), and at 250 °C, it limits columbite decomposition and, consequently, the release of SO_3_, which is not favorable for metal sulfate formation (eqs 1–4). However, similar results were obtained at 350 °C, where acid decomposition is favored, with the release of SO_3_. At 450 °C (higher than the boiling point), on the other hand, part of the released SO_3_ may volatilize and be lost before reacting with the solid matrix, showing lower leaching efficiencies. It is recommended higher sulfation temperatures to decrease impurities leaching (such as Fe, Th, Ca, and P), reduce sulfation time and reduce equipment damage.^34^ In our work, this approach has a disadvantage due to low leaching efficiencies in comparison to the use of KHSO_4_, and an in-depth explanation is needed in future studies. The increase of H_2_C_2_O_4_ concentration also benefited the leaching efficiency, and the limit based on acid solubility is 1.0 mol/L (Figure 6C).

**Figure 6:**
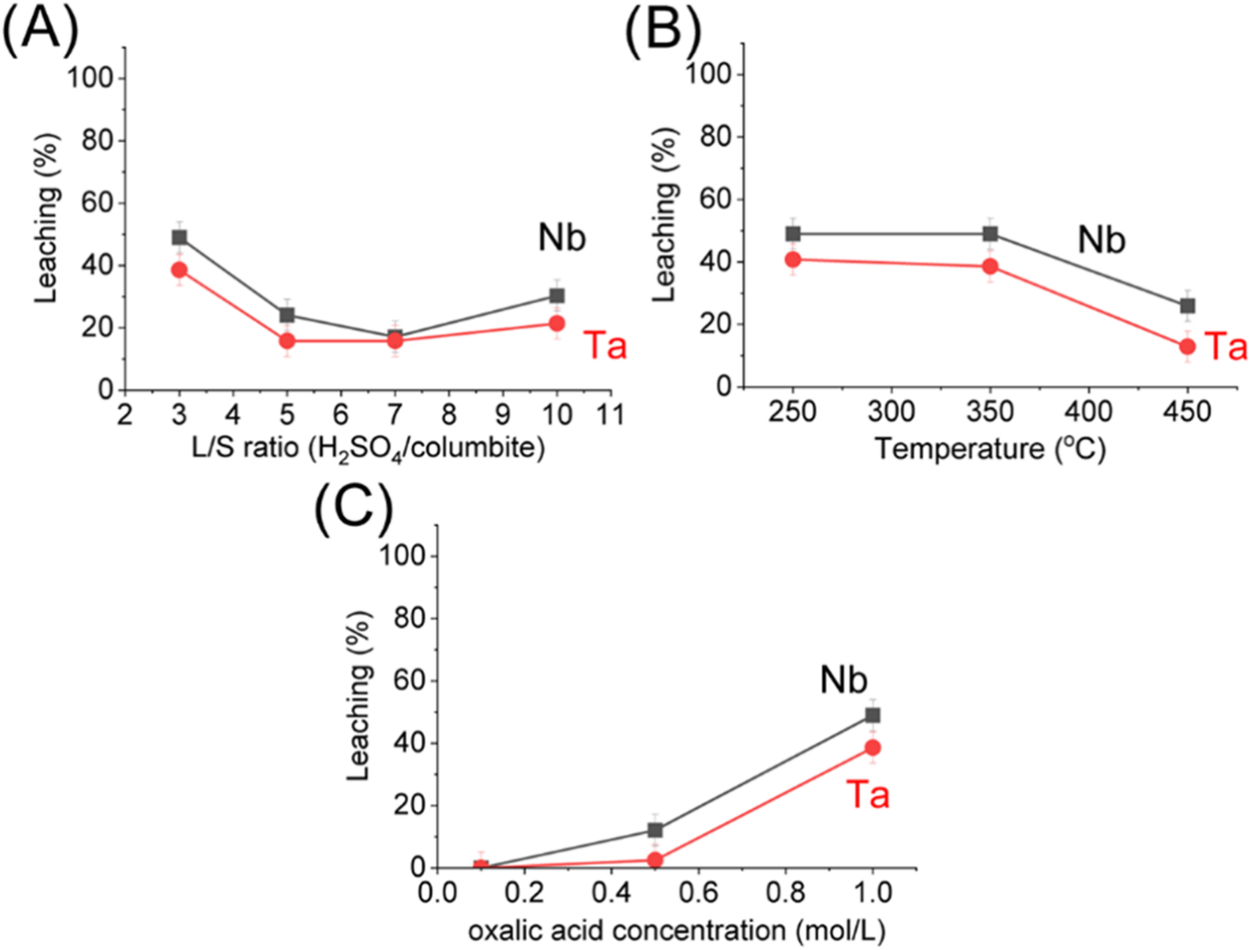
(A) Effect of the S/L ratio between columbite and H_2_SO_4_ during thermal treatment for 2 h at 350 °C on leaching with 1.0 mol/L H_2_C_2_O_4_ at a L/S ratio of 5 for 8 h at 90 °C. (B) Effect of the pretreatment temperature with analytical-grade H_2_SO_4_ at a L/S ratio of 3 for 2 h on leaching with 1.0 mol/L H_2_C_2_O_4_ at a L/S ratio of 5 for 8 h at 90 °C. (C) Effect of acid concentration on leaching with H_2_C_2_O_4_ at a L/S of 5 for 8 h at 90 °C, followed by thermal pretreatment with H_2_SO_4_ at a L/S ratio of 3 for 2 h at 350 °C.

Thermal pretreatment with KHSO_4_ yielded the best leaching performance, achieving 92.8% for Nb (3.48 g/L) and 88.4% for Ta (0.45 g/L) (Table S3). This was accomplished under optimal pretreatment conditions: an S/S ratio (columbite/KHSO_4_) of 3 at 650 °C for 3 h, followed by leaching with 1.0 mol/L H_2_C_2_O_4_ at a L/S ratio of 10 at 90 °C for 8 h. The results demonstrate that KHSO_4_ sulfation is a technically viable alternative to H_2_SO_4_, although a full assessment requires consideration of critical economic factors.

### 3.3 Preliminary Economic Analysis

A preliminary economic analysis is important for decision-making in hydrometallurgical processing. Our analysis considered the costs of reagents, energy (electricity) consumption, and potential value of Nb and Ta oxides in a leaching solution (without considering the separation/purification steps) for 1 kg of columbite processing (Table S4). The cost of KHSO_4_ (about 80%) represents the main issue for the process. If KHSO_4_ is replaced with H_2_SO_4_ (as in the processing of rare earth elements), it would represent a 9-fold reduction. Another drawback is the difficulty of recycling KHSO_4_; after the thermal treatment and leaching, K ions remained in the solution, while sulfate could be recycled in the thermal treatment step or after hydrometallurgical processing, which is easy to perform in H_2_SO_4_ processing. As a result, the process is not economically feasible without KHSO_4_ recycling, resulting in costs of 102–126 USD per kg of columbite and potential economic value in the leaching solution of 23.31 USD per kg of columbite. In our preliminary analysis, economic attractiveness is reached with the recycling of KHSO_4_ and H_2_C_2_O_4_ of 90–95%. Electricity costs (considering the US scenario = 0.12 USD/kWh) are relatively low, even considering high temperatures in thermal treatment (650 °C). We recommend that future studies focus on recycling KHSO_4_ and H_2_C_2_O_4_ in columbite processing to achieve economic feasibility. Technical advances were achieved, and our study consolidates them.

## 4 Conclusions

In this study, thermal treatments with KHSO_4_ and H_2_SO_4_ of columbite were evaluated for organic acid leaching to obtain Nb and Ta. Different from previous studies, including those of the research group, we evaluated the leaching of Nb and Ta from columbite using organic acid. The sample contains D90 of 154.8 μm, D50 of 79.7 μm, and D10 of 37.3 μm, mostly composed of FeNb_2_O_6_ (ferrocolumbite), SiO_2_ (quartz), UO_2_ (uranium oxide), and Pb_1.5_Nb_2_O_6.5_ (pyrochlore). The concentrations of Nb and Ta in columbite were about 23% and 3%, respectively. Low leaching rates of Nb and Ta were observed in the direct leaching reaction due to the strong acid resistance of FeNb_2_O_6_ and Pb_1.5_Nb_2_O_6.5_, with higher efficiencies using organic acid (H_2_C_2_O_4_) than using inorganic acid (H_2_SO_4_). Sulfation thermal treatment aimed at converting the refractory Nb and Ta minerals into more soluble compounds for leaching, where SO_3_ reacts with Nb and Ta oxides present in ferrocolumbite to form sulfates such as NbO­(SO_4_) and TaO­(SO_7_). The columbite/KHSO_4_ ratio of 1:3 resulted in the highest leaching efficiency with H_2_C_2_O_4_, reaching 94% for Nb and 80% for Ta. This indicates that in this case, even lower proportions of KHSO_4_ are sufficient to promote the formation of soluble sulfates. The increase of H_2_SO_4_ in sulfation has almost no effect on Nb and Ta leaching, although it is better than direct acid leaching. Temperature in sulfation plays a pivotal role due to the H_2_SO_4_ boiling point (337 °C). Under pretreatment conditions, the S/S ratio 3 (columbite/KHSO_4_) at 650 °C for 3 h followed by leaching with 1.0 mol/L H_2_C_2_O_4_, L/S ratio 10 at 90 °C for 8 h, leaching of Nb and Ta reached 92.8% and 88.4%, respectively. The process is not economically feasible without KHSO_4_ recycling in 90–95%. Our recommendation for future studies is that they focus on recycling KHSO_4_ and H_2_C_2_O_4_ in columbite processing to achieve economic feasibility.

## Supplementary Material



## Notes

Parameters evaluated in direct leaching experiments; leaching efficiency for direct leaching and after thermal treatment using H_2_SO_4_ and H_2_C_2_O_4_; and cost of the chemical reagents and electricity for preliminary economic assessment (PDF)
