# Supplementary material for: Leaching of Columbite Using an Organic Acid to Obtain Nb and Ta: Effects of Sulfate Thermal Treatment on Hydrometallurgical Processing
Source: ACS Omega. 2026 Mar 17;11(12):18961–9. doi: 10.1021/acsomega.5c11374 (PMC13044654; doi:10.1021/acsomega.5c11374)
Supplement: Supplementary file 1 [file ao5c11374_si_001.pdf]

Leaching of columbite using organic acid to obtain Nb and Ta: effect of sulfate thermal treatment for hydrometallurgical processing

Danielly Cristina da Silva Lucio[1=]; Thamyres Cardoso de Carvalho[2=]; Bárbara da Rocha Pereira[1]; Denise Crocce Romano Espinosa[1]; Jorge Alberto Soares Tenório[1]; Amilton Barbosa Botelho Junior[3,4] *.

[1] Department of Chemical Engineering, Polytechnical School, University of São Paulo, São Paulo, SP, 05508-080, Brazil.
[2] Department of Chemical Engineering, FEI University, São Bernardo do Campo, SP, 09850-901, Brazil.
[3] Department of Chemical Engineering, Norwegian University of Science and Technology, Trondheim, 7491, Norway.
** corresponding author: amilton.b.b.junior@ntnu.no; [=] equal first authors.*

SUPPLEMENTARY MATERIAL

Table S1: Parameters evaluated in direct leaching tests with $H_2C_2O_4$.

S/L ratio	1/3; 1/5; 1/7; 1/10
Acid concentration (mol/L)	0.1; 0.5 and 1mol/L
Temperature (°C)	25°C; 45°C; 60°C and 90°C
Time	1h; 2h; 5h and 8h

Table S2: Effect of the S/L ratio on the direct leaching of columbite with 1.0mol/L $H_2C_2O_4$ or 2.0mol/L H_2SO_4 for 8 h at 90°C.

	L/S ratio ($H_2C_2O_4$)				L/S ratio (H_2SO_4)	
	**3**	**5**	**7**	**10**	**5**	**10**
Nb	16.3%	40.0%	29.3%	58.9%	0.4%	0.9%
Ta	0.2%	9.7%	22.0%	29.1%	0.0%	0.2%
Fe	50.6%	68.6%	37.8%	96.3%	51.4%	49.4%
Pb	4.5%	10.1%	6.2%	2.5%	0.1%	0.1%
Si	0.0%	2.4%	0.0%	8.2%	0.7%	1.4%
U	30.8%	48.7%	25.2%	89.5%	17.2%	22.1%
Th	2.7%	3.2%	2.1%	1.8%	11.6%	70.5%
Zr	0.1%	0.9%	3.1%	2.3%	9.3%	9.1%
Hf	0.0%	15.0%	0.0%	25.0%	4.3%	4.3%

Table S3: Leaching efficiency and concentration of the leaching solution after pretreatment with $KHSO_4$ at an S/S ratio of 1:3 for 3h at 650°C and leaching 1.0mol/L $H_2C_2O_4$ at L/S ratio 3 for 8h at 90°C

Elements	Leaching efficiency	Concentration on the leaching solution (mg/L)
Nb	92.8%	3475.6
Ta	88.4%	444.5
Fe	97.9%	1395.5
Pb	8.4%	175
Si	0.0%	
U	88.4%	693.7
Th	0.0%	0
Zr	24.2%	88.5
Hf	0.0%	0

Table S4: Cost of the chemical reagents and electricity used in the process

	**Cost**
Nb_2O_5	59 USD/kg
Ta_2O_5	170 USD/kg
$KHSO_4$	27.52 USD/kg
$H_2C_2O_4$	7.97 USD/kg
Electricity	0.12 USD/kWh

Energy consumption = 2kW-10kW furnace.

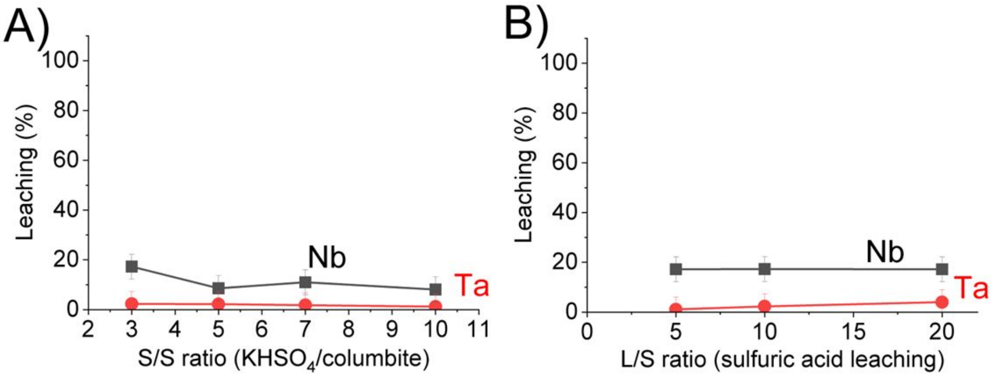

Figure S1: (a) Effect of the S/S ratio between columbite and $KHSO_4$ during thermal treatment for 3h at 650°C on the leaching with 1.0mol/L H_2SO_4 at an S/L ratio of 1:10 for 8 h at 90°C. (b) Effect of the S/L ratio of the treated sample/acid on leaching with 1.0mol/L H_2SO_4 for 8 h at 90°C, after pretreatment with $KHSO_4$ at an S/S ratio of 1:3 for 3h at 650°C.

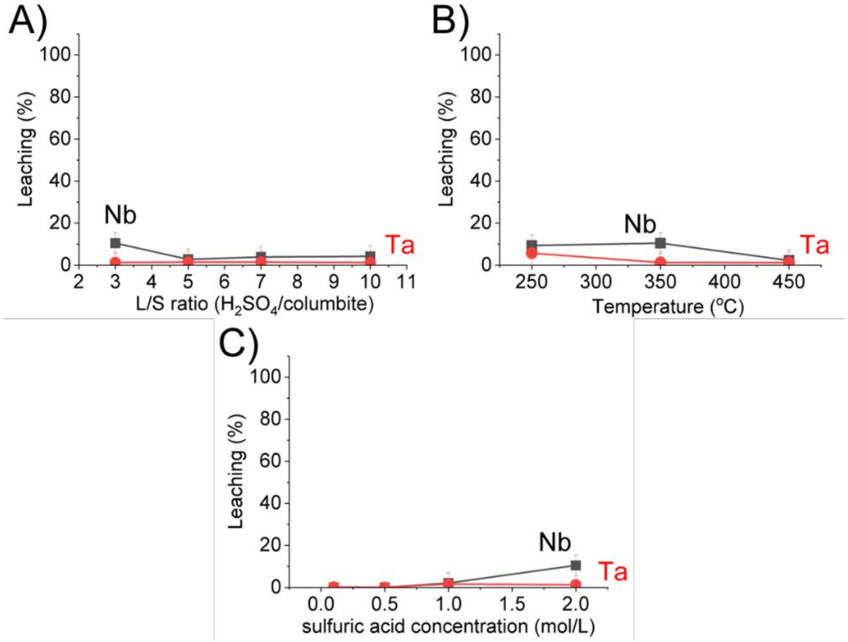

Figure S2: (a) Effect of the S/L ratio between columbite and H_2SO_4 during thermal treatment for 2 h at 350°C on leaching with 1.0mol/L H_2SO_4 at an S/L ratio of 1:5 for 8 h at 90°C. (b) Effect of the pretreatment temperature with analytical-grade H_2SO_4 at a 1:3 ratio for 2 h on leaching with 1.0mol/L H_2SO_4 at a 1:5 ratio for 8 h at 90°C. (c) Effect of acid concentration on leaching with H_2SO_4 at S/L 1:5, for 8 h at 90 °C, followed by thermal pretreatment with H_2SO_4 at a solid-to-liquid ratio of 1:3, for 2 h at 350 °C.
